# How to improve the human brucellosis surveillance system in Kurdistan Province, Iran: reduce the delay in the diagnosis time

**DOI:** 10.4178/epih.e2020058

**Published:** 2020-08-10

**Authors:** Meysam Olfatifar, Seyed Mehdi Hosseini, Payam Shokri, Soheila Khodakarim, Naghmeh Khadembashi, Sajjad Rahimi Pordanjani

**Affiliations:** 1 Gastroenterology and Liver Diseases Research Center, Research Institute for Gastroenterology and Liver Diseases, Shahid Beheshti University of Medical Sciences, Tehran, Iran; 2 Social Determinants of Health Research Center, Research Institute for Health Development, Kurdistan University of Medical Sciences, Sanandaj, Iran; 3 Department of Epidemiology, School of Allied Medical Sciences, School of Public Health and Safety, Shahid Beheshti University of Medical Sciences, Tehran, Iran; 4 English Language Department, School of Allied Medical Sciences, Shahid Beheshti University of Medical Sciences, Tehran, Iran; 5 Student Research Committee, Department of Epidemiology, School of Public Health and Safety, Shahid Beheshti University of Medical Sciences, Tehran, Iran

**Keywords:** Spatial analysis, Brucellosis, Exponential scanning statistics, Iran

## Abstract

**OBJECTIVES:**

Spatial information makes a crucial contribution to enhancing and monitoring the brucellosis surveillance system by facilitating the timely diagnosis and treatment of brucellosis.

**METHODS:**

An exponential scan statistic model was used to formalize the spatial distribution of the adjusted delay in the diagnosis time of brucellosis (time between onset and diagnosis of the disease) in Kurdistan Province, Iran. Logistic regression analysis was used to compare variables of interest between the clustered and non-clustered areas.

**RESULTS:**

The spatial distribution of clusters of human brucellosis cases with delayed diagnoses was not random in Kurdistan Province. The mean survival time (i.e., time between symptom onset and diagnosis) was 4.02 months for the short spatial cluster, which was centered around the city of Baneh, and was 4.21 months for spatiotemporal clusters centered around the cities of Baneh and Qorveh. Similarly, the mean survival time for the long spatial and spatiotemporal clusters was 6.56 months and 15.69 months, respectively. The spatial distribution of the cases inside and outside of clusters differed in terms of livestock vaccination, residence, sex, and occupational variables.

**CONCLUSIONS:**

The cluster pattern of brucellosis cases with delayed diagnoses indicated poor performance of the surveillance system in Kurdistan Province. Accordingly, targeted and multi-faceted approaches should be implemented to improve the brucellosis surveillance system and to reduce the number of lost days caused by delays in the diagnosis of brucellosis, which can lead to long-term and serious complications in patients.

## INTRODUCTION

Brucellosis is an endemic zoonotic disease in Iran [1] and many other countries, and is among the most important challenges impeding the economic development of many of these counties. Kurdistan Province, with an average annual incidence of 56.14 per 100,000 population between 2009 and 2016, is a province in Iran with a high rate of human brucellosis [2,3]. Human brucellosis can affect any organ or tissue and has a variety of signs and symptoms [4], including fever, fatigue, nausea, headache, weight loss, splenomegaly, and arthritis, which can overlap with the symptoms of many other diseases [5].

The lack of typical clinical manifestations can lead to misdiagnosis and/or a delayed diagnosis [6]; the consequent delays in treatment result in a chronic course of disease with long-term complications and difficult management. Thus, given the debilitating consequences of this disease, which can cause serious harm and even death, it should be diagnosed in a timely manner, since timely diagnoses approaches are an effective strategy for the prevention [6] and treatment of diseases [7] such as brucellosis.

However, regarding the absence of typical manifestations, as discussed above [3,8], and the fact that brucellosis is strongly influenced by socioeconomic and environmental [9] factors, access to additional information regarding the geographic distribution and epidemiological features of disease may enhance our ability to diagnose brucellosis [6] and strengthen the framework of our monitoring capability [10]. In Kurdistan Province, most brucellosis patients have a history of contact with livestock [2] (most of them are males, villagers, farmers and ranchers); furthermore, having an understanding of the type of infected livestock (cattle, sheep, and goats are the most common types of infected animals in Iran [3]), livestock vaccination coverage, and the type of bacterial strains (*Brucella melitensis* and *B. abortus* are the most common strains in Iran) in the region can assist in making more rational decisions to combat the disease. Furthermore, spatial analysis can also be used to assess the accessibility of healthcare services [10], an important factor in health-seeking behaviors of individuals with brucellosis, with potential impacts on delays in diagnosis. In this regard, an exponential scan statistic model for the clustering of brucellosis cases with delayed diagnoses was used to provide auxiliary information in order to improve the brucellosis surveillance system in Kurdistan Province.

## MATERIALS AND METHODS

### Basic information

Data on brucellosis cases were obtained from the Vice-Chancellor of Health Affairs of the Kurdistan University of Medical Sciences from January 2007 to December 2016. As individual-level variables, age, sex (male or female), residence (rural or urban), contact with livestock (yes or no), high-risk occupation (yes or no), and type of disease (new or old) were selected as covariate variables or nuisance factors, and the time interval between onset and diagnosis of brucellosis was considered as the independent or time-to-event variable. Patients who received confirmation of human brucellosis within 30 days from the onset of manifestations were classified as having a timely diagnosis, while those who received a confirmation of brucellosis more than 30 days from the onset of manifestations were classified as having a delayed diagnosis. Only patients with a delayed diagnosis were selected for analysis. Cases with incomplete information or missing date information were excluded. Cities were used as the units of analysis. Farmers, ranchers, veterinarians, slaughterhouse workers, butchers, and other related workers were considered as individuals with high-risk occupations.

### Geographical analysis

The clusters of brucellosis cases with delayed diagnoses were evaluated by applying the exponential scan statistic model using the SaTScan version 9.6 (https://www.satscan.org/). Before applying the scan statistic, the survival time (time between onset and diagnosis) was adjusted for the effect of nuisance factors using the exponential regression model. The delayed diagnosis time was adjusted for contact with livestock products and variables regarding the type of disease that remained significant variables in the exponential regression model in Stata version 14 (StataCorp., College Station, TX, USA). Thus, the following formula was used for adjustment:

(1) $$t_{i}^{adj}\;=\;t_{i}\;\times \;\exp\;[-\overset{p}{\underset{j=2}{\sum {\hat{\beta }}_{j}}}\;\times \;(x_{ij}\;-\;k_{j})]\;$$

Where, *k_j_* is min (*x_ij_*), ${\hat{\beta }}_{j}\;$ is the estimated regression coefficient, *t_i_* is the observed survival time, and *i* and *j* indicate the number of cases and number of covariates included in the model. More information about this adjustment technique can be obtained from the literature [11]. Subsequently, the spatial and spatiotemporal scan statistics were applied to identify clusters with long survival (i.e., areas with a longer survival time than found outside of the clustered areas) and short survival (i.e., areas with a shorter survival time than found outside of the clustered areas). In terms of brucellosis, a shorter survival time (the difference between onset and diagnosis time) is better because it indicates a timely and sensitive surveillance system.

The survival probability graph was added to the maps of cases inside and outside of each corresponding cluster using Kaplan-Meier estimates in R version 3.6.0 (https://www.r-project.org/). The same color was used for detected clusters and their corresponding survival curves.

Finally, logistic regression analysis was used to obtain a deeper understanding regarding the distinction of cluster areas with other areas in terms of age, sex (male or female), high-risk occupation (yes or no), disease history in other family members (yes or no), and areas covered by livestock vaccination (yes or no). In this analysis, only the short survival clusters were considered due to the similarity of the long survival clusters (spatiotemporal and spatial clusters) to each other, as well as the time period of the spatiotemporal clusters, which approximately corresponded to the middle of the study period. The patients forming a cluster were considered cases (coded as 1) and subsequently, other patients were considered as controls (coded as 0). Univariate logistic regression was used to select the candidate variables (variables with p<0.25) to perform multivariable logistic regression. The results of the regression analysis were reported separately for each of the short survival clusters and for the sum of identified clusters in each case.

### Clinical description and case definition

Although brucellosis does not have any specific symptoms, the most common and important symptoms of the disease in humans are fever, chills, sweating, body and muscle pain, and joint and spine pain.

In the Iranian brucellosis surveillance system, the following definitions and criteria are used to diagnose brucellosis:

Suspected case: Presence of clinical signs compatible with brucellosis with an epidemiological association with infected livestock or livestock products; Probable case: A suspected case with a Rose Bengal test titer ≥ 1/80; Definitive case: A suspected or probable case in whom *Brucella* has been isolated from clinical specimens or who has an agglutinating antibody titer higher than 1/40 or a quadruple or a sharp increase in agglutinating antibody levels 2 weeks after the initial test.

### Ethics statement

This project was ethically approved by the Iran National Committee for Ethics in Biomedical Research (No. IR.SBMU.PHNS. REC.1398.030).

## RESULTS

### Spatial survival clusters

**Figure 1.**
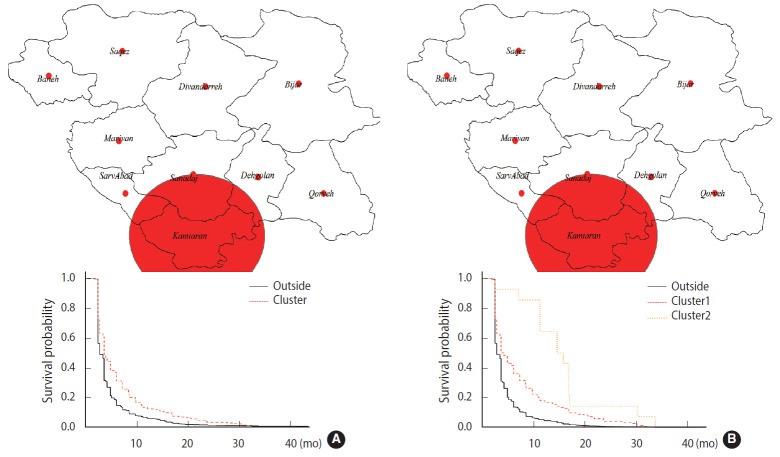
Long spatial (A) and spatiotemporal (B) survival clusters of brucellosis cases with delayed diagnoses and their corresponding graphs.

**Figure 2.**
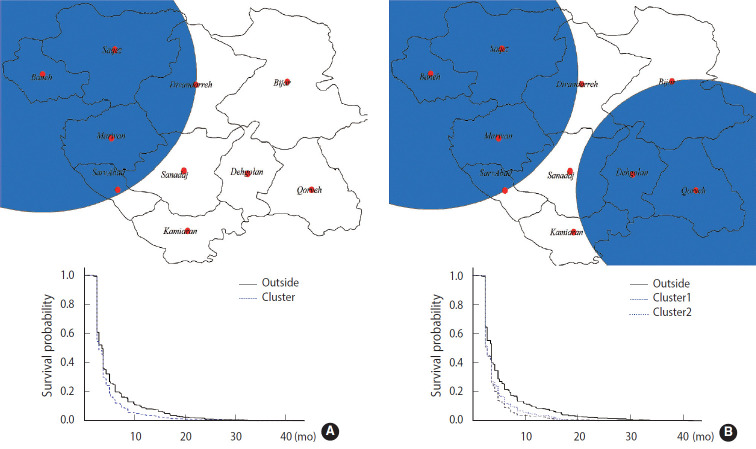
Short spatial (A) and spatiotemporal (B) survival clusters of brucellosis cases with delayed diagnoses and their corresponding graphs.

Geographical studies on survival, as a health outcome, are of interest [11]. Hence, the exponential scan statistic was used to identify clusters of brucellosis cases, as described above. A short survival cluster centered on the city of Baneh was detected, along with a long survival cluster centered on the city of Kamyaran, by applying the spatial scan statistic (Table 1). The mean survival time of the short and long survival clusters was equal to 4.02 months and 6.56 months, respectively; both of these values are very high, potentially leading to serious complications (Figures 1A and 2A). Likewise, the spatiotemporal scan statistic was applied and 2 short survival clusters centered on the cities of Baneh and Qorveh and 2 long survival clusters centered on the cities of Kamyaran and Bijar were detected (Table 1). The mean survival time of the short survival clusters was 3.90 months and 4.21 months, respectively. Similarly, the mean survival time of the long survival time clusters was 7.44 months and 15.69 months, respectively (Figures 1A and 2A). The time interval of the short survival clusters was from January 2013 to December 2016, while the time intervals for the long survival clusters were from January 2011 to December 2014 and January 2007 to December 2008, respectively. Overlapping the time interval of the short survival clusters with those of the long survival clusters, and considering the fact that the short survival cluster time was aligned with the final years of the study, it can be inferred that the delay in diagnosis has been reduced, but still remains at an average of 3.84 months.

These results reveal that the distribution of brucellosis cases with delayed diagnoses was not random in Kurdistan Province, suggesting the need to periodically assess and enhance the surveillance system [12].

### Comparison of cluster areas with other areas

Multiple pieces of evidence have demonstrated differences between clustered and non-clustered areas in terms of several associated factors. Hence, logistic regression analysis was applied to determine the potential differences in the clustered and non-clustered areas. Accordingly, the variables of age, residence, and living in areas covered by livestock vaccination were associated with the formation of short survival spatial clusters. Specifically, the cases who lived in areas covered by animal vaccination had nearly 3.5 times (odds ratio [OR], 3.24; 95% confidence interval [CI], 2.53 to 4.15) higher odds of being in the cluster, while rural residents had lower odds (OR, 0.59; 95% CI, 0.40 to 0.85) (Table 2). For the 2 short spatiotemporal survival clusters (Figures 1B and 2B), lower odds were associated with having a low-risk occupation (OR, 0.60; 95% CI, 0.40 to 0.90) and living in areas covered by livestock vaccination (0.48; 95% CI, 0.38 to 0.61), while having family members with a positive history of the disease was associated with higher odds of being in these clusters (OR, 1.40; 95% CI, 1.05 to 1.85). For the first spatiotemporal cluster, cases who lived in areas covered by animal vaccination had nearly 2 times (OR, 1.93; 95% CI, 1.46 to 2.57) higher odds of being in the cluster (Table 2).

Likewise, for the second spatiotemporal cluster, cases who were male (OR, 1.28; 95% CI, 1.01 to 1.63), lived in rural areas (OR, 1.34; 95% CI, 0.84 to 2.16) and had high-risk jobs (OR, 2.45; 95% CI, 1.56 to 3.86) had higher odds of being in the cluster. Nonetheless, dwellers in areas covered by livestock vaccination (OR, 0.27; 95% CI, 0.21 to 0.35) had lower odds of being in the cluster (Table 2).

These results indicate underlying differences between the clustered and non-clustered areas, even among the short spatial and spatiotemporal clusters.

## DISCUSSION

Overall, our results underscore the need to improve the brucellosis surveillance system in Kurdistan Province through better monitoring and timely diagnosis of brucellosis, as the spatial distribution of the cases with delayed diagnoses was not random. Even the 3 short survival clusters that were observed in the cities of Baneh and Qorveh had an average survival time of 4.04 months, which can lead to long-term consequences. The time interval of the spatiotemporal short survival clusters was from 2013 to 2016, indicating the continued delay in the diagnosis of brucellosis. A significant difference was also observed between the clustered and non-clustered areas in terms of the studied variables. This may imply that demographic and personal factors can also play a role in the timely diagnosis of brucellosis beyond the performance of the surveillance system. However, some potential problems in the performance of the surveillance system may also play a role, including a lack of workforce and financial resources, failure to collect the reporting forms on time, inattention or carelessness of the treatment staff in completing the reporting forms, and lack of adequate training about the disease and its symptoms.

In line with the present study, Kazerooni et al. [12] reported that underreporting occurred in 41.8% of cases, with an average delay of 56.5 days timeliness (time elapsed between different levels of the surveillance system) for the diagnosis of brucellosis cases in Iran’s Communicable Diseases Surveillance System. Nejat et al. [13] also showed that the delays and sensitivity of brucellosis surveillance systems were 58 days and 12.1%, respectively. It can be concluded that there is an urgent need to improve the surveillance system for zoonotic diseases such as brucellosis, at least in some provinces, to reduce the incidence of long-term complications.

It was observed that males and cases with high-risk occupations had higher odds of being in the clusters, which aligned with our expectations because multiple lines of evidence [14-16] have shown that brucellosis has a higher prevalence among males and people with high-risk jobs, such as ranchers [14], butchers [17] and slaughterhouse workers [17,18]. That is, these groups are likely to be reckless towards the disease and its symptoms, which may cause them to postpone seeking healthcare services, thereby increasing their odds of be included in the clusters. Hence, these groups should be considered in targeted strategies for enhancing the surveillance system.

Likewise, it was observed that people with a positive family history of brucellosis were more likely to be in the clusters. This is reasonable and is supported by other studies. A screening study on endemic areas in Iran [19] revealed that the family members of brucellosis cases were at an elevated risk of being infected and that screening of family members could lead to early diagnoses of the disease. Therefore, screening of household members in clustered areas to enhance the brucellosis surveillance system may be advisable.

Vaccination of livestock is one of the best strategies for controlling brucellosis [7,20,21]; however, two different patterns were observed, as being in an area covered by livestock vaccination was associated with higher odds (OR, 3.24; 95% CI, 2.53 to 4.15) of being in the short survival spatial cluster and lower odds (OR, 0.48; 95% CI, 0.38 to 0.61) of being in the two short survival spatiotemporal clusters (Tables 1 and 2). This difference between the units forming the clusters can probably be explained in terms of control and prevention approaches and consequently, the inherent characteristics (demographic, socioeconomic, and personal factors) of the people in the detected clusters. Another justification may be rooted in the misreporting of vaccination coverage in the clustered areas across time, especially for the short spatial cluster, and/or low vaccination effectiveness. These results also indicate that clusters with different constituent units should not be combined, as doing so may lead to bias.

Meanwhile, several pieces of evidence have shown that being affected with brucellosis is associated with living in rural areas [22,23], and subsequently a delay in diagnosis. A different pattern was observed in terms of residence for the spatial cluster (lower odds of being in the cluster) and the second spatiotemporal cluster (higher odds of being in the cluster). The explanation proposed above likely holds true for this finding; in other words, this finding reflects differences between the units forming the clusters and consequently in the performance of the surveillance system. Consequently, appropriate strategies must be used with respect to these differences to improve the surveillance system.

All in all, the brucellosis surveillance system in Kurdistan Province should be improved through a targeted and multi faceted approach due to the long delays in diagnoses and the presence of different spatial patterns in this province. However, it was not possible to clarify the true reason for delays in the brucellosis diagnosis, because such delays can be rooted in patients’ lack of adequate knowledge about brucellosis and its symptoms, delays in health-seeking behaviors [24], and negligence towards the clinical symptoms, as well as the inadequate performance of the brucellosis surveillance system in terms of collection and reporting processes. Hence, further and more detailed studies are required to evaluate the performance of the surveillance system.

## Figures and Tables

**Table 1.** Spatial and spatiotemporal survival clusters of brucellosis cases with delayed diagnoses in Kurdistan Province

Clusters ID			Locations	No. of cases	Cluster cases/total no. of cases	Mean survival time (mo)	Radius (km)	Log likelihood ratio	p-value
Spatial clusters									
	Short survival								
		1	Ba, Sa, Ma, Sar, Di	633	0.425	4.02	101.65	7.59	0.001
	Long survival								
		1	Ka, San	218	0.146	6.56	44.23	13.52	0.001
Spatiotemporal clusters									
	Short survival								
		1	Ba, Sa, Ma, Sar	279	0.187	3.90	98.62	7.27	0.002
		2	Qo, De, Bi	455	0.306	4.21	81.11	6.00	0.013
	Long survival								
		1	Ka, San	156	0.104	7.44	44.23	19.34	0.001
		2	Bi	14	0.009	15.69	0.00	15.20	0.001

Ba, Baneh; Sa, Saqez; Ma, Marivan; Sar, Sarvabad; Di, Divandareh; Ka, Kamyaran; San, Sanadaj; Qo, Qorveh; De, Dehgolan; Bi, Bijar.

**Table 2.** Logistic regression analysis for clusters of brucellosis cases with delayed diagnoses in Kurdistan Province

Variables	OR (95% CI)	p-value	aOR (95% CI)	p-value
Spatial cluster				
Age	0.99 (0.98, 0.99)	0.039	0.99 (0.98, 0.99)	0.038
Residence		0.185		0.005
Urban	1.00 (reference)		1.00 (reference)	
Rural	1.22 (0.90, 1.65)		0.59 (0.40, 0.85)	
Livestock vaccination		0.001		0.001
No	1.00 (reference)		1.00 (reference)	
Yes	2.79 (2.24, 3.48)		3.24 (2.53, 4.15)	
History of disease		0.048		0.373
No	1.00 (reference)		1.00 (reference)	
Yes	1.30 (1.00, 1.69)		1.13 (0.85, 1.50)	
Spatiotemporal clusters				
Sex		0.162		0.202
Female	1.00 (reference)		1.00 (reference)	
Male	1.15 (0.94, 1.41)		1.15 (0.92, 1.44)	
Residence		0.018		0.412
Urban	1.00 (reference)		1.00 (reference)	
Rural	1.43 (1.06, 1.92)		1.20 (0.77, 1.86)	
Livestock vaccination		0.001		0.001
No	1.00 (reference)		1.00 (reference)	
Yes	0.60 (0.48, 0.74)		0.48 (0.38, 0.61)	
History of disease		0.051		0.018
No	1.00 (reference)		1.00 (reference)	
Yes	1.29 (0.99, 1.69)		1.40 (1.05, 1.85)	
High-risk occupation		0.001		0.016
Yes	1.00 (reference)		1.00 (reference)	
No	1.77 (1.34, 2.33)		1.65 (1.09, 2.47)	
First spatiotemporal cluster				
History of disease		0.028		0.126
No	1.00 (reference)		1.00 (reference)	
Yes	1.42 (1.03, 1.94)		1.28 (0.93, 1.77)	
Livestock vaccination		0.001		0.001
No	1.00 (reference)		1.00 (reference)	
Yes	1.95 (1.48, 2.58)		1.93 (1.46, 2.57)	
Second spatiotemporal cluster				
Sex		0.004		0.039
Female	1.00 (reference)		1.00 (reference)	
Male	1.39 (1.11, 1.73)		1.28 (1.01, 1.63)	
Residence		0.034		0.203
Urban	1.00 (reference)		1.00 (reference)	
Rural	1.44 (1.02, 2.02)		1.34 (0.84, 2.16)	
High-risk occupation		0.001		0.001
No	1.00 (reference)		1.00 (reference)	
Yes	1.97 (1.42, 2.74)		2.45 (1.56, 3.86)	
Livestock vaccination		0.001		0.001
No	1.00 (reference)		1.00 (reference)	
Yes	0.34 (0.27, 0.43)		0.27 (0.21, 0.35)	

OR, odds ratio; CI, confidence interval; aOR, adjusted odds ratio.
